# Antioxidant procyanidin B2 protects oocytes against cryoinjuries via mitochondria regulated cortical tension

**DOI:** 10.1186/s40104-022-00742-y

**Published:** 2022-08-16

**Authors:** Qingrui Zhuan, Jun Li, Xingzhu Du, Luyao Zhang, Lin Meng, Yuwen Luo, Dan Zhou, Hongyu Liu, Pengcheng Wan, Yunpeng Hou, Xiangwei Fu

**Affiliations:** 1 Key Laboratory of Animal Genetics, Breeding and Reproduction of the Ministry of Agriculture and Rural Affairs, Key Laboratory for Animal Genetic Improvement, College of Animal Science and Technology, National Engineering Laboratory for Animal Breeding, China Agricultural University, Beijing, China; 2 Department of Reproductive Medicine, Reproductive Medical Center, The First Hospital of Hebei Medical University, Shijiazhuang, Hebei China; 3 State Key Laboratories of Agrobiotechnology, College of Biological Sciences, China Agricultural University, Beijing, China; 4 State Key Laboratory of Sheep Genetic Improvement and Healthy Breeding, Institute of Animal Husbandry and Veterinary Sciences, Xinjiang Academy of Agricultural and Reclamation Sciences, Shihhotze, China

**Keywords:** Cortical tension, Meiosis, Mitochondria, Oocytes, Procyanidin B2, Vitrification

## Abstract

**Background:**

Irreversible cryodamage caused by oocyte vitrification limited its wild application in female fertility preservation. Antioxidants were always used to antagonist the oxidative stress caused by vitrification. However, the comprehensive mechanism underlying the protective role of antioxidants has not been studied. Procyanidin B2 (PCB2) is a potent natural antioxidant and its functions in response to vitrification are still unknown. In this study, the effects of PCB2 on vitrified-thawed oocytes and subsequent embryo development were explored, and the mechanisms underlying the protective role of PCB2 were systematically elucidated.

**Results:**

Vitrification induced a marked decline in oocyte quality, while PCB2 could improve oocyte viability and further development after parthenogenetic activation. A subsequent study indicated that PCB2 effectively attenuated vitrification-induced oxidative stress, rescued mitochondrial dysfunction, and improved cell viability. Moreover, PCB2 also acts as a cortical tension regulator apart from strong antioxidant properties. Increased cortical tension caused by PCB2 would maintain normal spindle morphology and promote migration, ensure correct meiosis progression and finally reduce the aneuploidy rate in vitrified oocytes. Further study reveals that ATP biosynthesis plays a crucial role in cortical tension regulation, and PCB2 effectively increased the cortical tension through the electron transfer chain pathway. Additionally, PCB2 would elevate the cortical tension in embryo cells at morula and blastocyst stages and further improve blastocyst quality. What’s more, targeted metabolomics shows that PCB2 has a beneficial effect on blastocyst formation by mediating saccharides and amino acids metabolism.

**Conclusions:**

Antioxidant PCB2 exhibits multi-protective roles in response to vitrification stimuli through mitochondria-mediated cortical tension regulation.

**Supplementary Information:**

The online version contains supplementary material available at 10.1186/s40104-022-00742-y.

## Introduction

Oocyte cryopreservation could prolong, protect and secure female fertility [1], which has lead to a worldwide interest in oocyte cryopreservation [2]. Vitrification is an ultrafast cooling method performed with a very high concentration cryoprotectant for dehydration to avoid the formation of ice crystals during oocyte cryopreservation, which ultimately leads to higher cell survival, fertilization, embryo development and pregnancy rates compared with traditional slow-freezing [3–6]. However, studies reveal that impaired oolemma permeability [5], oxidative stress [7] and the toxicity of cryoprotectants during vitrification [8] can still cause damages to the oocytes [9, 10], especially deteriorating cytoskeleton [11] and mitochondrial function [12, 13], which eventually obstruct subsequent embryo development and need further study [14–16]. It is reported that mechanical properties play a pivotal role in oocyte development [17]. Since vitrified oocyte experiences dramatic shrinking and swelling during dehydration and rehydration, it is imperative to explore the mechanical variations.

Generally, the layer beneath the cell surface is considered to be the cortex, and the thickness of cell cortex varies depending on the cell type [18]. Oocyte cortex behaves as a function unit, not only in regulating the mechanical properties [18–20], but also in modulating polar body formation, cellular asymmetry creation and the egg-to-embryo transition [21–23]. Cortical tension reflects long time-scale mechanics, therefore can be considered as a sensitive readout of cortical cytoskeleton contractility [19]. In oocytes, non-muscle myosin II and Ezrin/Radixin/Moesin (ERM) protein family have a great contribution to oocyte cortical tension regulation [24–26]. ERM functions in the active phosphorylated-ERMs (pERM) form [26]. Active pERMs are localized in the oocyte cortical, declined during meiotic maturation to MII and then increased after fertilization, reflecting the dynamic changes in tension during these developmental transitions [24]. Similarly, myosin-II mediated cortical tension is regulated by phosphorylation of its regulatory light chain (pMRLC) [27]. It was reported that exclusion of myosin-II from the cortex induced a decline in the cortical tension, and the subsequent increased concentration of myosin-II in the cytoplasm would eventurally lead to meiosis defects [28]. Thus, the level of pERM and cortical pMRLC is consistent with the change of cortical tension, while cytoplasmic pMRLC is negatively corrlerated with cortical tension [24, 28, 29]. Up to now, the changes of cortical tension in vitrified oocytes are still unclear.

As an important organelle in developmental regulation during oogenesis and early embryogenesis, mitochondria can influence redox-sensitive biological activities and redox-dependent signaling pathways [30, 31]. The morphology, distribution and ultrastructure of mitochondria were impaired after vitrification [32, 33]. Although mitochondrial oxidative phosphorylation (OXPHOS) provides a special metabolic chamber to produce ATP, it can cause collateral cell damage by releasing reactive oxygen species (ROS) [34]. It is acknowledged that oxidative stress is one of the main factors attributed to the decreased quality of vitrified oocytes. Increased ROS levels arising from the vitrification procedure are the major sources of oxidative stress [35–37]. ROS can attack proteins, DNA, cell membrane, and also microtubules, thereby disrupting oocyte structure and function [36, 38]. Since vitrified oocytes are under oxidative stress, antioxidants are introduced in conventional cryo-solutions to protect oocytes from cryoinjuries [5, 39–42]. N-acetylcysteine [40] can improve the quality of mature mouse oocytes after vitrification, melatonin [5] can enhance the efficiency of human oocytes’ cryopreservation and resveratrol [39] improves the development of vitrified bovine embryos. Procyanidins, a class of naturally occurring plant polyphenols with strong antioxidant properties, play beneficial roles in metabolic diseases and inflammatory response [43–46]. The dimer procyanidin B2 [4,8′-BI- [( +)-epicatechin]] (PCB2) is a member of oligomeric anthocyanins precursors and its anti-oxidative effect is more potent than other oligomers [47–52]. The effect of PCB2 on the oocytes viability under vitrification-induced oxidative stress and the mechanism underlying is not determined yet.

In this study, the effect of PCB2 on mitochondrial function (membrane potential, ATP, Ca^2+^ homeostasis) and the relationship between mitochondrial production and cortical tension regulation were first studied in vitrified oocytes. The dramatic changes brought by vitrification resulted in meiosis defects and mitochondrial dysfunction and ultimately led to the quality decline in vitrified oocytes. Apart from antioxidant properties, PCB2 induced elevated cortical tension via improving mitochondrial function. Those results may provide a new angle to understand the effect of vitrification on oocytes and give hints to improving current vitrification techniques.

## Materials and methods

### Animals and housing

Studies were performed using 8-week-old female mice (CD-1® (ICR)) (Vital River Laboratory Animal Technology Co., Ltd. Beijing, China). Mice were housed in ventilated cages on a 12 h light/12 h dark cycle (lights on from 08: 00 to 20: 00) under controlled temperature (22 ± 2 °C) with food and water freely available.

### Chemicals and antibodies

All chemicals and drugs were purchased from Sigma (St. Louis, MO, USA) unless otherwise indicated. The anti-H2A.X antibody, anti-LC3 antibody, anti-rabbit IgG (H + L), F (ab’)_2_ Fragment (Alexa Fluor® 594 Conjugate) antibody, anti-pERM antibody, anti-pMRLC antibody were purchased from Cell Signaling Technology (Danvers, MA, USA). The anti-CDX2 antibody was purchased from BioGenex (San Francisco, USA). The anti-Nanog antibody was purchased from Abcam (Cambridge, United Kingdom). The anti-alpha Tubulin antibody was purchased from Thermo Fisher (Shanghai, China). The Fluorescein (FITC)–conjugated Affinipure Goat Anti-Rabbit IgG (H + L) secondary antibody was purchased from Proteintech (Beijing, China).

### Oocyte collection and parthenogenesis activation

Germinal vesicle (GV) stage oocytes were collected after superovulated by 10 IU pregnant mare serum gonadotropin (PMSG, Ningbo Shusheng Veterinary Drug Co., Ltd., Ningbo, China). After 48 h, cumulus-oocyte complexes (COCs) were obtained and cumulus cells were removed by repeatedly mouth pipetting, then oocytes were cultured in M16 medium at 37 °C with 5% CO_2_. Germinal vesicle breakdown (GVBD) and polar body extrusion (PBE) rate were recorded at 2 h and 12 h, respectively.

To collect in vivo matured MII oocytes, mice were superovulated using 10 IU PMSG, followed by injection with 10 IU human chorionic gonadotrophin (hCG, Ningbo Shusheng Veterinary Drug Co., Ltd., Ningbo, China) 48 h later to induce superovulation. At 13–14 h post-hCG injection, ovulated oocytes were retrieved from the ampulla and collected in M2 medium, the cumulus cells were removed enzymatically using 0.1% (w/v) hyaluronidase. PCB2 (5 μg/mL), concanavalin A (ConA, 10 μg/mL) and ML-7 (30 μmol/L) were added according to the purpose of the experiment. For parthenogenesis activation, denuded oocytes were transferred first into (Ca^2+^)-free human tubal fluid (HTF) medium supplemented with 10 mmol/L strontium chloride and 5 μg/mL cytochalasin B (Merck, Darmstadt, Germany), incubated at 37 °C with 5% CO_2_ for 2.5 h. Then oocytes were transferred into HTF with 5 μg/mL cytochalasin B, incubated at 37 °C with 5% CO_2_ for 3.5 h. Activated oocytes were then cultured in KSOM medium at 37 °C with 5% CO_2_ for early embryo development. Cleavage and blastocyst rates were recorded at 24 h and 96 h, respectively.

### Oocyte vitrification and thawing

For vitrification, pretreatment solution was PBS medium contained 10% (v/v) dimethylsulfoxide (DMSO) and 10% (v/v) ethylene glycol (EG) while vitrification solution was PBS medium contained 30% Ficoll (w/v), 15% EG (v/v) and 15% DMSO (v/v) in 0.5 mol/L sucrose. GV and in vivo matured MII oocytes were vitrified by the open pulled straws method, respectively. Vitrified oocytes were stored in LN_2_ for at least 1 week. For thawing, the oocytes were rinsed in 0.5 mol/L sucrose for 5 min, then rinsed three times in M2 medium. After thawing, GV oocytes were in vitro matured as mentioned above, MII oocytes were further recovered for 1 h and then used for subsequent experiment.

### Immunofluorescence (IF) staining and confocal microscopy

Oocytes/embryos were fixed with 4% (w/v) paraformaldehyde (PFA) for 40 min at room temperature, followed by permeabilization with 0.5% Triton X-100 at room temperature for 1 h. After being blocked in 3% BSA for 1 h at room temperature, oocytes/embryos were incubated with different primary antibodies (anti-CDX2, 1:500; anti-Nanog, 1:1000; anti-γH2A.X, 1:100; anti-LC3, 1:100; anti-α-tubulin, 1:8000; anti-pERM, 1:600; anti-pMRLC, 1:300) overnight at 4 °C. The oocytes/embryos were further incubated with an appropriate secondary antibody for 1 h at room temperature. Finally, all oocytes/embryos were stained with 4′,6-diamidino-2-phenylindole (DAPI, Vector Laboratories, Burlingame, CA, USA) for 5 min and the fluorescent images were taken with laser scanning confocal microscopy (A1 Cell Imaging System; Nikon, Tokyo, Japan) under the same staining procedure and confocal microscopy parameters.

For F-actin staining, oocytes/embryos were fixed with 4% (w/v) paraformaldehyde (PFA) for 40 min at room temperature, followed by permeabilization with 1% Triton X-100 at room temperature for 1 h. After being blocked in 3% BSA for 1 h at room temperature, oocytes/embryos were incubated with Phalloidin-Teramethyl-rhodamine B overnight at 4 °C. After washing three times, all oocytes/embryos were stained with DAPI for 5 min and the fluorescent images were taken with laser scanning confocal microscopy (A1 Cell Imaging System; Nikon, Tokyo, Japan) under the same staining procedure and confocal microscopy parameters.

To conduct chromosome spread, the zona pellucida was removed by 0.5% pronase. Then oocytes were fixed in a medium of 1% paraformaldehyde in distilled H_2_O containing 0.15% Triton X-100 and 3 mmol/L dithiothreitol. After air drying, the chromosome was stained with DAPI for 10 min. Samples were examined under a laser scanning confocal microscope.

For mitochondrial membrane potential (MMP) quantification, oocytes were measured with JC-1 assay kit (Beyotime, Shanghai, China). Briefly, denuded oocytes were incubated with 10 mmol/L JC-1 at 37 °C with 5% CO_2_ for 20 min. Then, oocytes were washed with M2 three times and observed under a laser scanning confocal microscope or fluorescence microscope (IX73, Olympus, Tokyo, Japan). The MMP was calculated as the ratio of red fluorescence, corresponding to activated mitochondria (J-aggregates), to green fluorescence, corresponding to less activated mitochondria (J-monomers).

For active mitochondrial distribution assay, oocytes were incubated in M2 medium containing 5 μmol/L Mito-Tracker (Beyotime, Shanghai, China) for 20 min. Then oocytes were washed with M2 medium three times and analyzed using a confocal laser scanning microscope.

The active mitochondrial temperature assay was determined using the thermosensitive mitochondrial‐targeted fluorescent dye Mito Thermo Yellow (MTY), as described previously [53]. MTY was first added to the prewarmed culture medium and incubated at 37 °C in 5% CO_2_ for 15 min. Then, oocytes were added to the medium and the samples were incubated at 37 °C in 5% CO_2_ for another 15 min. Oocytes were washed with M2 medium three times and analyzed using a confocal laser scanning microscope.

Subcellular Ca^2+^ was evaluated using Fluo 3-AM, Rhod 2-AM and Fluo 4-AM to indicate the intracellular calcium, mitochondrial calcium ([Ca^2+^]_m_) and endoplasmic reticulum calcium ([Ca^2+^]_ER_), respectively. The zona pellucida of oocytes was removed by 0.5% pronase and then oocytes were incubated with dye for 20 min at 37 °C with 5% CO_2_. Oocytes were washed with M2 medium three times and analyzed using a confocal laser scanning microscope.

Mean fluorescence intensity per unit area within the region of interest was used to quantify the fluorescence of each oocyte/embryo. Fluorescence intensity was assessed using NIS-Elements AR software (Nikon Instruments, Tokyo, Japan).

### Determination of ATP levels

Cellular ATP content was measured using an Enhanced ATP Assay Kit (Beyotime, Shanghai, China). The ATP detection method was optimized according to previous reports [54, 55]. Briefly, serial dilutions of ATP were prepared (from 0 to 40 pmol). A total of three biological replicates were performed with 10 oocytes per replicate. Denuded oocytes for each group were collected in a centrifuge tube containing 20 μL lysis buffer and homogenized by vortexing until lysis. ATP assay buffer, standard solutions and ATP detection diluent were injected into each well, and luminescence activity was measured immediately using a luminometer (Infinite F200; Tecan Austria GmbH, Austria). ATP content was calculated using a standard curve. The total amount of ATP was divided by the number of oocytes in each sample to obtain the mean content per oocyte (pmol/oocyte).

### Intracellular ROS and GSH level assay

Denuded oocytes were added to the medium which contains 1 mmol/L 2′,7′-dichlorofluorescin diacetate (2',7'-DCFHDA) for measuring ROS or 10 μmol/L Cell Tracker Blue (Invitrogen, Carlsbad, CA, USA) for measuring GSH at 37 °C in 5% CO_2_ for 20 min. Then oocytes were washed by M2 three times. The fluorescence was examined under a fluorescence microscope (IX73, Olympus, Tokyo, Japan) with a filter at 460-nm excitation for ROS and 370-nm excitation for GSH. The fluorescence of each oocyte was analyzed by EZ-C1Free-Viewer (Nikon, Tokyo, Japan).

### Annexin-V staining of oocytes

Oocytes were stained with an Annexin-V staining kit (Vazyme, Nanjing, China) according to the manufacturer’s instructions. Briefly, oocytes were stained for 10 min with 100 μL of binding buffer containing 5 μL Annexin-V-FITC at 37 °C. Samples from each group were mounted on glass slides and fluorescent signals were analyzed by a fluorescence microscope (IX73, Olympus, Tokyo, Japan).

### Real-time quantitative PCR (qRT-PCR)

After thawing and further recovery for 1 h, 50 MII oocytes per repelicate were collected from different groups and stored in the -80 ℃. Total RNA was extracted using an RNeasy micro-RNA isolation kit (Qiagen, Valencia, CA, USA) and then it was reversed to cDNA using a QuantiTect Reverse Transcription Kit (Qiagen). Primers for the published reference RNA sequences for real-time quantitative polymerase chain reaction (qPCR) were listed in Table 1. Primers were tested for efficiency to ensure their specificity. qPCR was performed by adding 1 μL cDNA to a mixture of SYBR premix qPCR SuperMix (Qiagen), forward and reverse primers (10 μmol/L), and RNase-free water to a final volume of 20 μL. The cycling conditions were 94 °C for 30 s, followed by 42 cycles of 94 °C for 5 s and 60 °C for 34 s. Relative mRNA levels of target genes were calculated using the 2^–ΔΔCt^ method with *Ppia* and *Rpl7* as the reference genes according to previous reports [56, 57].

**Table 1:** Primer sequences used for quantitative real-time PCR

Gene	Primer sequence (5ʹ to 3ʹ)	NCBI reference sequences
*Beclin1*	F: ATGGAGGGGTCTAAGGCGTC. R: TCCTCTCCTGAGTTAGCCTCT	NM_001359819.1
*Map1lc3a*	F: CATGAGCGAGTTGGTCAAGA. R: TTGACTCAGAAGCCGAAGGT	NM_025735.3
*Ulk1*	F: AAGTTCGAGTTCTCTCGCAAG. R: CGATGTTTTCGTGCTTTAGTTCC	NM_001347394.1
*Atg14*	F: GAGGGCCTTTACGTGGCTG. R: AATAGACGAAATCACCGCTCTG	NM_172599.4
*Lamp1*	F: CAGCACTCTTTGAGGTGAAAAAC. R: ACGATCTGAGAACCATTCGCA	NM_001317353.1
*Lamp2*	F: TGTATTTGGCTAATGGCTCAGC. R: TATGGGCACAAGGAAGTTGTC	NM_001017959.2
*Mfn1*	F: GGACTTTATCCGAAACCAGA. R: TGAGATTGAAGAATGGAGGC	NM_024200.4
*Mfn2*	F: TTCTTGTGGTCGGAGGAGTG. R: CTTTGGTGGTCCAGGTCAGT	NM_001285920.1
*Opa1*	F: CCGAGGATAGCTTGAGGGTT. R: CGTTCTTGGTTTCGTTGTGA	NM_001199177.1
*Drp1*	F: CAGGTGGTGGGATTGGAGAC. R: CTGGCATAATTGGAATTGGTTT	NM_001025947.2
*Ppia*	F: GAGCTGTTTGCAGACAAAGTTC. R: CCCTGGCACATGAATCCTGG	NM_008907.2
*Rpl7*	F: TCAATGGAGTAAGCCCAAAG. R: CAAGAGACCGAGCAATCAAG	NM_011291.5

*Beclin1*, beclin 1, autophagy related; *Mpa1lc3a*, microtubule associated protein 1 light chain 3 alpha; *Ulk1*, unc-51 like autophagy activating kinase 1; *Atg14*, autophagy related 14; *Lamp1*, lysosomal associated membrane protein 1; *Lamp2*, lysosomal associated membrane protein 2; *Mfn1*, mitofusin 1; *Mfn2*, mitofusin 2; *Opa1*, mitochondrial dynamin like GTPase; *Drp1*, Dynamin related protein 1; *Ppia*, peptidylprolyl isomerase A; *Rpl7*, ribosomal protein L7

### Targeted metabolomics analysis

The amino acids and sugar in blastocyst culture were measured using ultra-performance liquid chromatography (UPLC). Samples were analyzed on AB SCIEX 5500 QQQ-MS system (SCIEX, Framingham, MA, USA) equipped with a Waters UPLC (Milford, MA, USA).

For the quantification of amino acids and sugar metabolites, 200 μL blastocyst culture supernatant for each group were collected and stored at -80 ℃ before determination. After slow thawing at 4 °C, 50 μL sample was removed and reconstituted with 450 μL of ice ethanol containing 100 ng/mL of internal standard. The mixture was allowed to stand at 4 °C for 30 min and centrifuged at 12,000 r/min for 10 min. The sample extracts were injected onto an Xbridge BEH Amide Column (4.6 mm × 150 mm, 3.5 μm), and the column temperature was maintained at 40 °C. The UPLC system employed a gradient elution program consisting of water with 0.1% formic acid (mobile phase A) and acetonitrile (mobile phase B). The gradient elution conditions were 0–2 min, 85% B; 2–7 min, 65% B; 7–10 min, 30% B; 10–12 min, 85% B, with a 0.45 mL/min flow rate. The retention times were shown in Additional file 1: Table S1. The relative amount of target metabolites were normalized to the peak area of the IS. Data were analyzed using MultiQuant software (SCIEX, Abingdon, United Kindom).

### Experimental design

This study mainly consisted of Exp. 1, 2, and 3. In each of the experiments, all the fresh oocytes were randomly divided into three groups: fresh group (control), vitrified without (vitrified group), or with PCB2 treatment (V + PCB2 group). V + PCB2 stands for PCB2 addition in both vitrification/warming and the recovery medium, and the concentration was 5 μg/mL. In terms of GV oocytes, three groups were assigned as mentioned above. PCB2 were supplemented with the same concentration of 5 μg/mL in the maturation medium. GV oocytes were cultured for 2 h to count GVBD and 12 h to count PBE.

In Exp. 1, vitrified-thawed MII oocytes were in vitro recovered for 1 h. After thawing, redox status, organelle distribution, mitochondrial function, Ca^2+^ level, DNA damage, early apoptosis and autophagy, changes of cortical tension (pERM and pMRLC) and underlying mechanisms were explored. Besides, genes related to mitochondrial fusion/fission (*Mfn1*, *Mfn2*, *Opa1* and *Drp1*) and autophagy (*Beclin1*, *Mpa1lc3a*, *Ulk1*, *Atg14*, *Lamp1*, *Lamp2*) were quantified by qPCR.

In Exp. 2, vitrified-thawed GV oocytes were transferred into M16 medium with/without PCB2 for in vitro maturation. The effect of PCB2 on oocyte meiosis progression during vitrification and the underlying mechanism was explored. GVBD and PBE rates were recorded in the three groups. Next, spindle morphology, aneuploidy rate and spindle positioning were detected.

In Exp. 3, the lasting effect of PCB2 on embryo development was studied. In this experiment, parthenogenesis activation was performed on vitrified-thawed in vivo MII oocytes. Firstly, the blastocyst quality was determined by CDX2 and Nanog staining. Next, cortical tension regulatory protein pERM was evaluated in different developmental stages (2-cell, 4-cell, 6- to 8-cell, morula, blastocyst) by immunostaining. Finally, targeted metabolomics analysis was performed using the day 4 culture media.

### Statistical analysis

All percentages or values from at least three repeated experiments were expressed as mean ± SEM, and the number of oocytes observed was labeled in parentheses as (n). Data were analyzed by unpaired-samples *t*-test, provided by GraphPad Prism 8 (GraphPad Software Inc., La Jolla, CA, USA) statistical software. The level of significance was accepted as *P* < 0.05.

## Results

### PCB2 improves oocyte viability and embryo development after vitrification

**Fig. 1:**
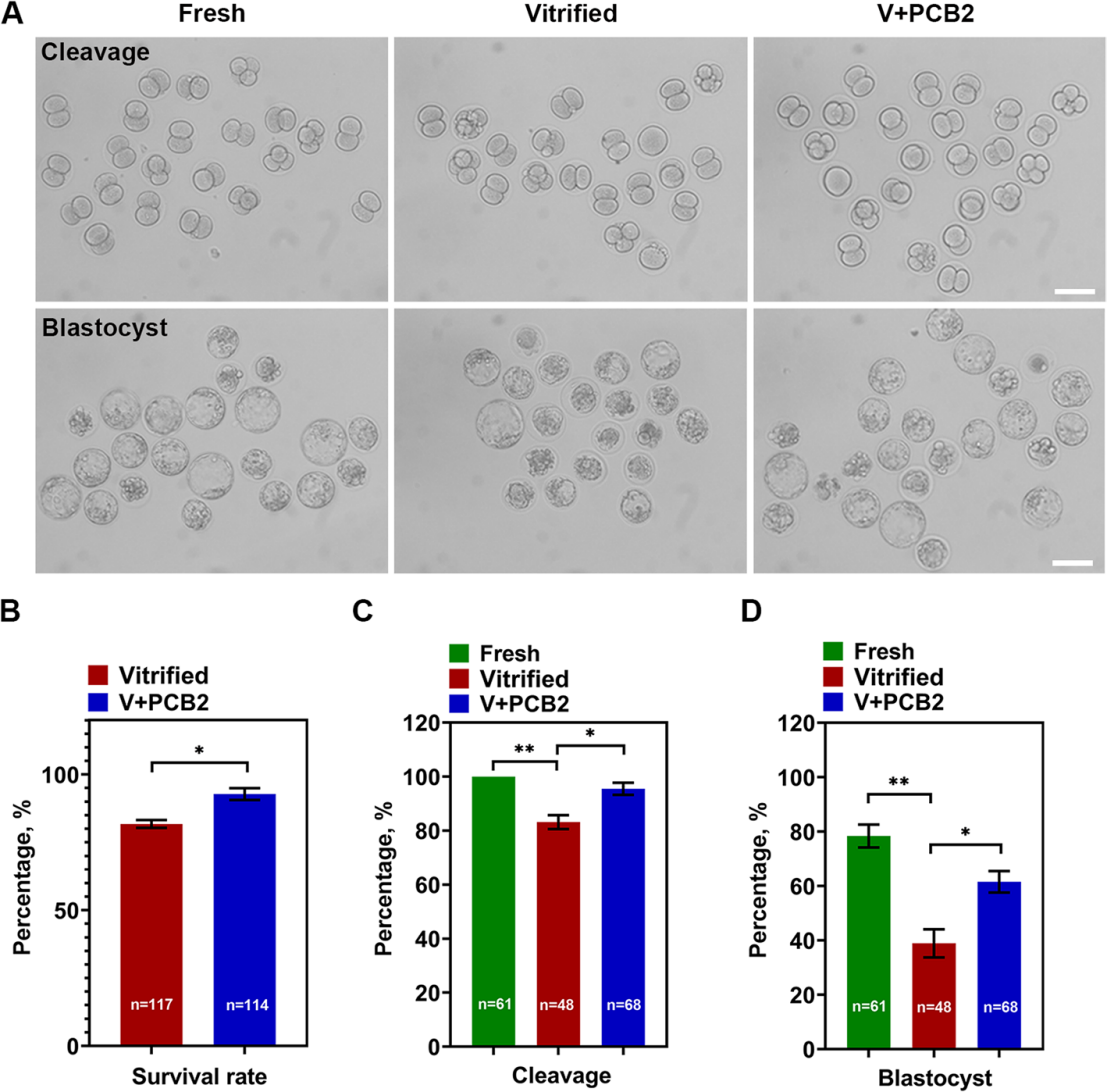
Survival rate and embryo development of vitrified-thawed MII oocytes. **A** Representative images of cleavage and blastocyst fromation. Scale bar = 50 μm. **B** The survival rate after thawing. **C** The rate of cleavage formation. **D** The rate of blastocyst formation. “n” represents the cell number used in this experiment. Data are presented as mean percentage (mean ± SEM) of at least three independent experiments. ^*^P < 0.05, ^**^P < 0.01, *ns* non significance

We first examined the survival rate of vitrified-thawed oocytes with or without PCB2 treatment. As shown in Fig. 1B, the survival rate of vitrified-thawed oocytes was increased after PCB2 treatment (Vitrified = 81.76 ± 1.44%, V + PCB2 = 92.79 ± 2.18%, *P* < 0.05). To explore the potential role of PCB2 in embryo development, oocytes from different groups were thawed and used for parthenogenetic activation. As shown in Fig. 1A, C and D, PCB2 could significantly improve the cleavage rate (Vitrified = 83.13 ± 2.58%, V + PCB2 = 95.48 ± 2.26%, *P* < 0.05) and blastocyst rate (Vitrified = 38.92 ± 5.18%, V + PCB2 = 61.51 ± 3.94%, *P* < 0.05) as compared to the non-supplemented group.

### Effects of PCB2 on oxidative stress and organelle distribution after vitrification

**Fig. 2:**
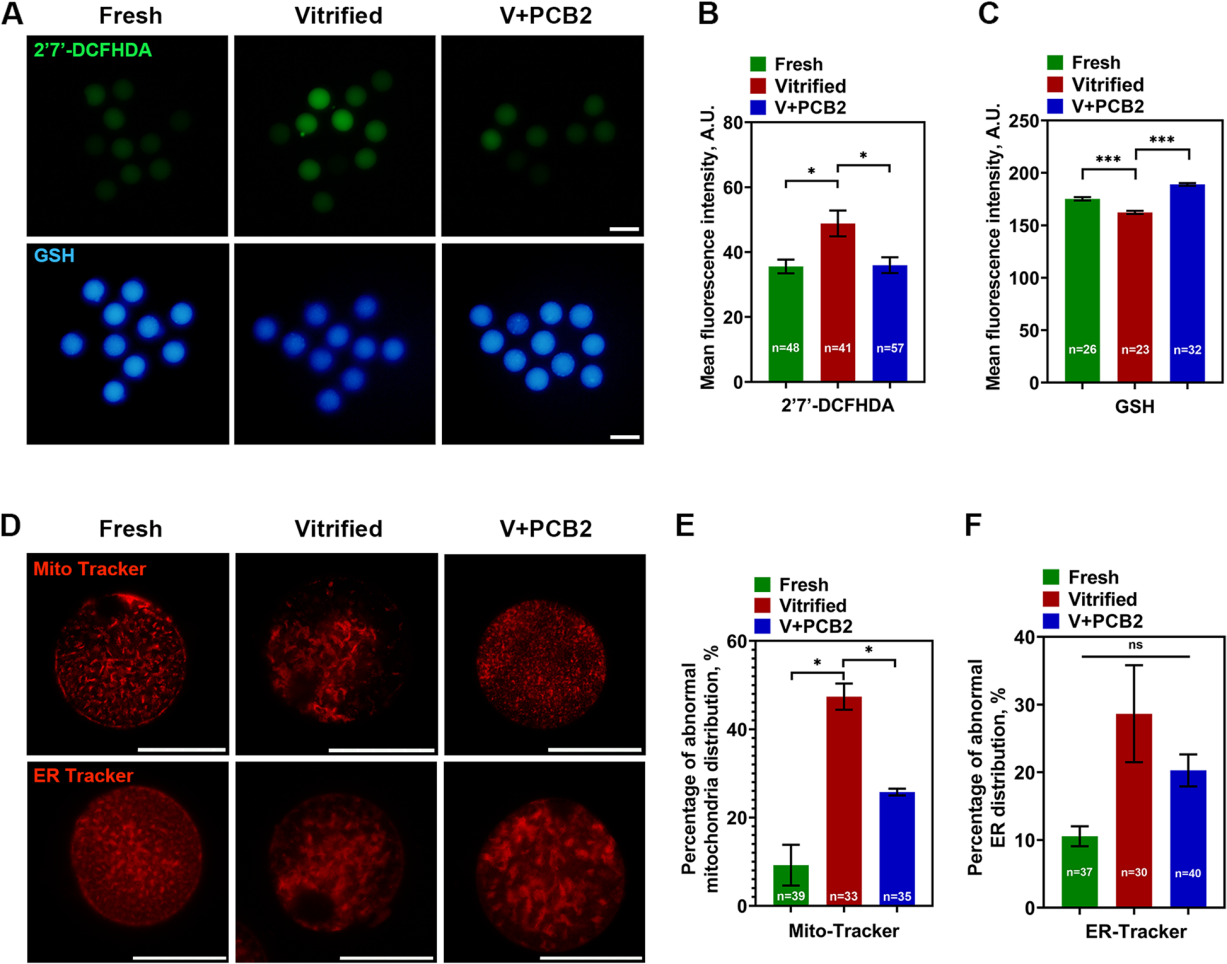
PCB2 can recover redox status and organelle distribution. **A** ROS and GSH levels of oocytes in different groups. Scale bar = 50 μm. **B** The fluorescence intensity of ROS signals. **C** The fluorescence intensity of GSH signals. **D** Representative images of mitochondria and ER distribution. Scale bar = 50 μm. **E** Rate of abnormal mitochondria distribution. **F** Rate of abnormal ER distribution. “n” represents the cell number used in this experiment. Data are presented as mean percentage (mean ± SEM) of at least three independent experiments. ^*^P < 0.05, ^***^P < 0.001, *ns* non significance

The redox state of the oocyte is important for the maintenance of cell viability. Therefore, ROS and GSH levels of oocytes were measured using 2',7'-DCFHDA and Cell Tracker Blue, respectively. As shown in Fig. 2A-C, PCB2 ameliorated vitrification-induced oxidative stress in oocytes, indicated by reduced ROS level (Vitrified = 48.80 ± 4.00, V + PCB2 = 35.94 ± 2.43, *P* < 0.05) and increased GSH level (Vitrified = 162.30 ± 1.49, V + PCB2 = 189.10 ± 1.23, *P* < 0.001). The distribution of mitochondria and ER were also measured. PCB2 could reduce the abnormal distribution of mitochondria (Vitrified = 47.40 ± 2.96%, V + PCB2 = 25.76 ± 0.76%, *P* < 0.05), but the ER distribution was not affected after vitrification (*P* > 0.05) (Fig. 2D-F).

### Mitochondria function was improved after PCB2 treatment in vitrified oocytes

**Fig. 3:**
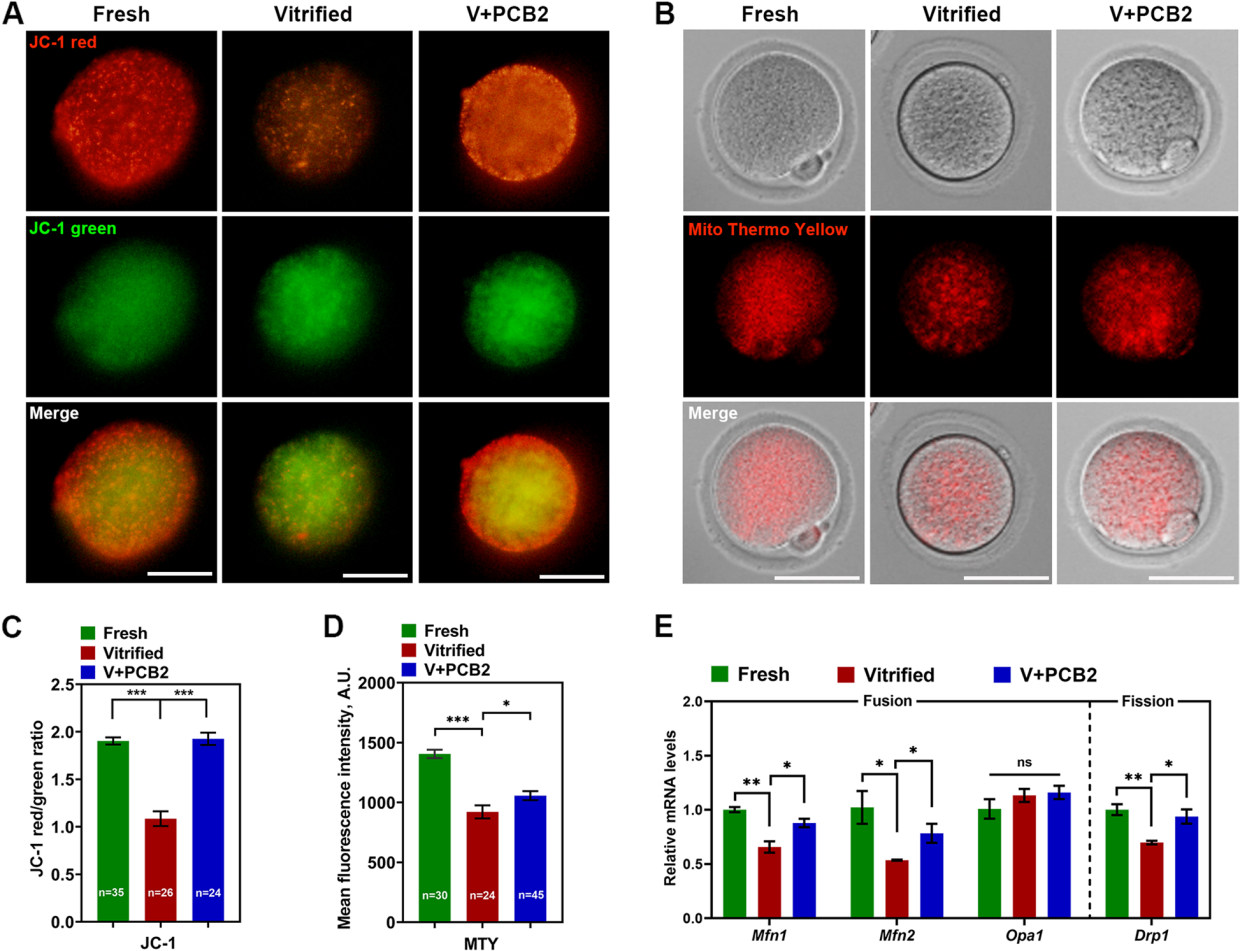
Mitochondrial function was impaired after vitrification. **A** Mitochondrial membrane potential was detected by JC-1 staining. Scale bar = 50 μm. **B** Mitochondrial temperature was detected by MTY fluorescent probe. Scale bar = 50 μm. **C** Quantification of the mitochondria membrane potential level. **D** Quantification of the MTY signals. **E** Expression of *Mfn1*, *Mfn2*, *Opa1* and *Drp1* was examined by qPCR. “n” represents the cell number used in this experiment. Data are presented as mean percentage (mean ± SEM) of at least three independent experiments. ^*^P < 0.05, ^**^P < 0.01, ^***^P < 0.001, *ns* non significance

Mitochondrial membrane potential can directly reflect the function of mitochondria. As shown in Fig. 3A and C, MMP was significantly decreased in vitrified oocytes, whereas PCB2 effectively reversed the reduction (Vitrified = 1.10 ± 0.08, V + PCB2 = 1.93 ± 0.06, *P* < 0.001). In addition, Mito Thermo Yellow (MTY) is a temperature-sensitive fluorescent probe, in which fluorescence intensity was negatively correlated with temperature. After vitrification, oocytes showed decreased fluorescence intensity, indicating that mitochondria temperature was significantly increased after vitrification, and PCB2 treatment reduced intracellular mitochondria temperature (Vitrified = 921.10 ± 54.83, V + PCB2 = 1056.00 ± 37.72, *P* < 0.05) (Fig. 3B and D). Genes related to mitochondrial fusion (*Mfn1*, *Mfn2* and *Opa1*) and fission (*Drp1*) were also examined. *Mfn1*, *Mfn2* and *Drp1* were all misexpressed in vitrified oocytes but restored after PCB2 supplementation (*P* < 0.05) (Fig. 3E).

### PCB2 restores mitochondrial and endoplasmic reticulum Ca^2+^ levels in vitrified oocytes

**Fig. 4:**
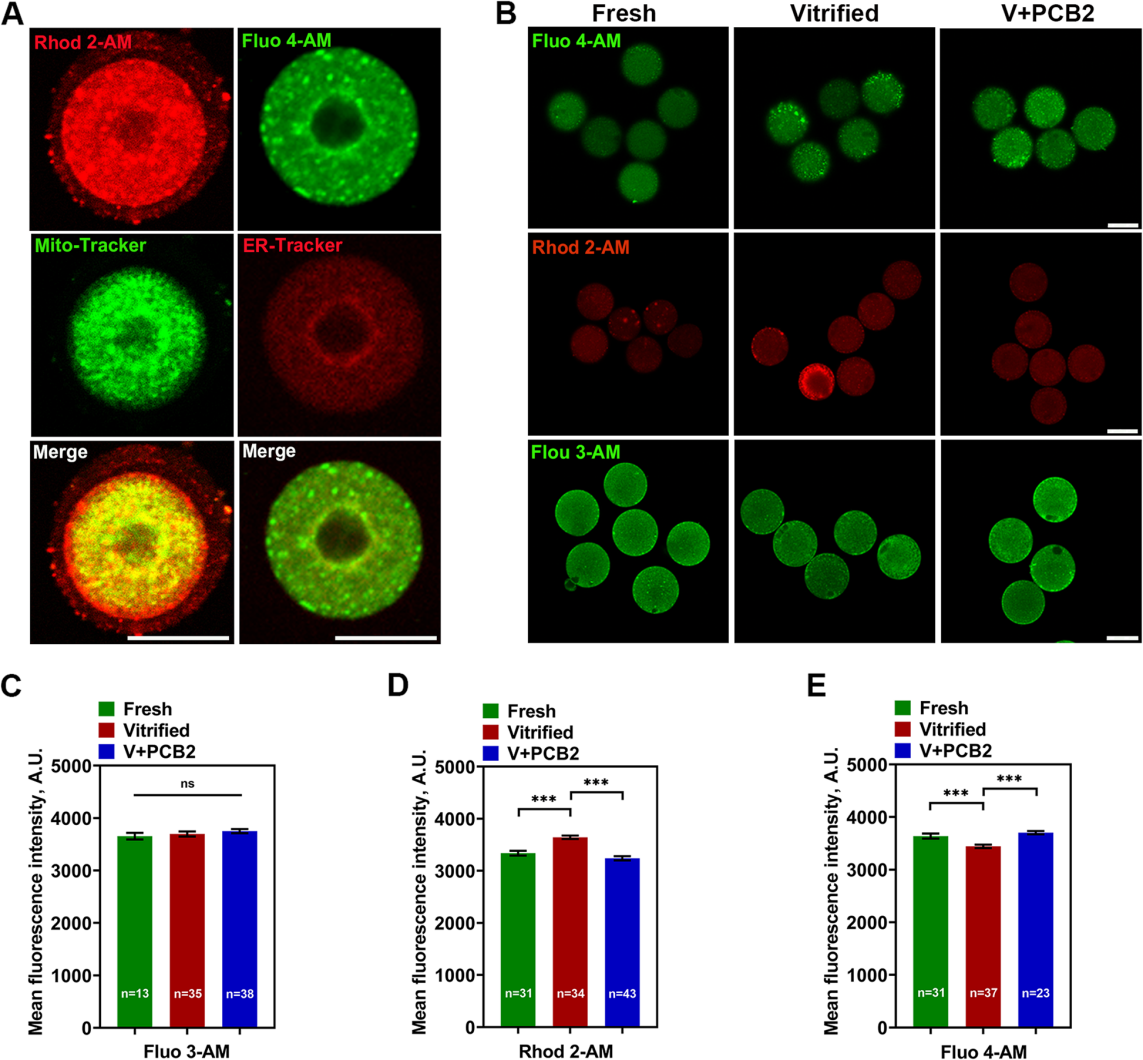
PCB2 has a positive role in regulating calcium level in vitrified oocytes. **A** Rhod 2-AM and Fluo 4-AM co-stained with Mito-tracker and ER-tracker respectively to indicate [Ca^2+^]_m_ and [Ca^2+^]_ER_. Scale bar = 50 μm. **B** Representative images of cytoplasmic calcium, [Ca^2+^]_m_,and [Ca^2+^]_ER_ in different groups. Scale bar = 50 μm. **C**-**E** Quantification of Fluo 3-AM, Rhod 2-AM and Fluo 4-AM fluorescence intensity. “n” represents the cell number used in this experiment. Data are presented as mean percentage (mean ± SEM) of at least three independent experiments. ^***^P < 0.001, *ns* non significance

Calcium homeostasis of oocytes plays a significant role in the subsequent development capacity, Fluo 3-AM, Rhod 2-AM, and Fluo 4-AM were used to detect the cytoplasmic calcium, [Ca^2+^]_m_, and [Ca^2+^]_ER_, respectively. To determine whether Rhod 2-AM and Fluo 4-AM can specifically track [Ca^2+^]_m_ and [Ca^2+^]_ER_ in oocytes, we used Mito-Tracker and ER-Tracker to co-stain with the above two calcium dyes in GV oocytes, respectively (Fig. 4A). The result showed that the fluorescent Ca^2+^ signal was localized within the corresponding organelle, declaring that Rhod 2-AM and Fluo 4-AM were efficient in tracking Ca^2+^ signals. After vitrification, [Ca^2+^]_m_ was significantly increased (Fresh = 3339.00 ± 45.39, Vitrified = 3643.00 ± 32.92, *P* < 0.001) while [Ca^2+^]_ER_ (Fresh = 3637.00 ± 48.65, Vitrified = 3443.00 ± 31.28, *P* < 0.001) was decreased. However, cytoplasmic calcium was not affected after vitrification. As expected, PCB2 supplementation effectively restored [Ca^2+^]_m_ (Vitrified = 3643.00 ± 32.92, V + PCB2 = 3242.00 ± 40.65, *P* < 0.001) and [Ca^2+^]_ER_ (Vitrified = 3443.00 ± 31.28, V + PCB2 = 3703.00 ± 30.51, *P* < 0.001) levels of vitrified-thawed oocytes.

### PCB2 inhibits DNA damage, apoptosis, and autophagy in vitrified oocytes

**Fig. 5:**
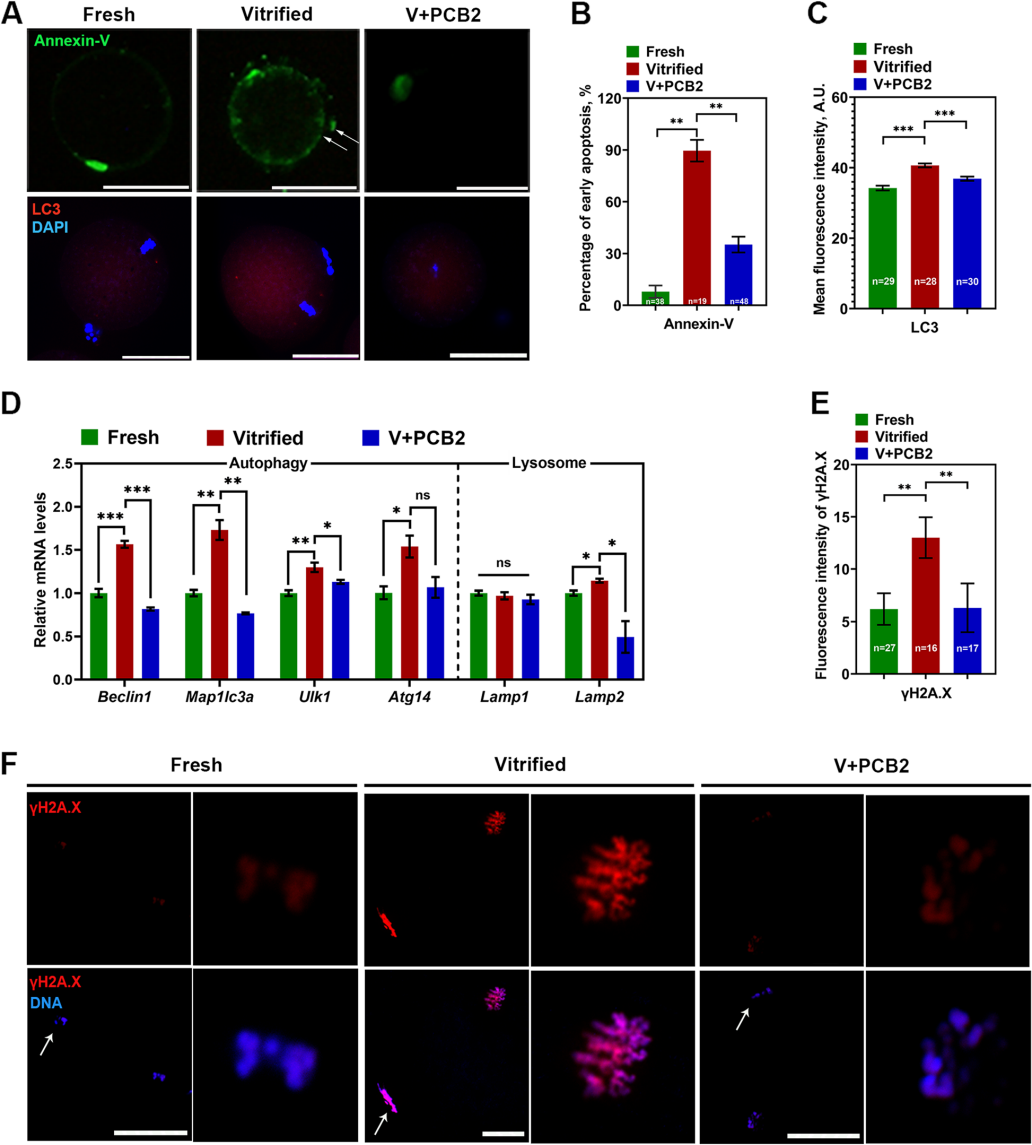
PCB2 can alleviate oxidative stress-related cell damage. **A** Representative images of early apoptotic indicator Annexin-V and autophagy indicator LC3 in different groups. DNA was counterstained with DAPI (blue). Scale bar = 50 μm. **B** The rate of early apoptosis. **C** The fluorescence intensity of LC3 signals. **D** Expression of *Beclin1*, *Map1lc3a*, *Ulk1*, *Atg14*, *Lamp1*, and *Lamp2* was examined by qPCR. **E** Relative fluorescence intensity of γH2A.X signals. **F** Representative images of DNA damage in different groups. Arrow indicates polar body. Scale bar = 50 μm. “n” represents the cell number used in this experiment. Data are presented as mean percentage (mean ± SEM) of at least three independent experiments. ^*^P < 0.05, ^**^P < 0.01, ^***^P < 0.001, *ns* non significance

Oxidative stress usually results in the accumulation of DNA damage and accelerates early apoptosis and autophagy. An Annexin-V probe was used to assess apoptosis initiation in oocytes (Fig. 5A-B). The increased percentage of early apoptosis in vitrified oocytes was suppressed by supplementation with PCB2 (Vitrified = 89.58 ± 6.25%, V + PCB2 = 35.21 ± 4.60, *P* < 0.01). Furthermore, vitrified-thawed oocytes showed more intense LC3 signals than fresh ones (Fresh = 34.23 ± 0.67, Vitrified = 40.64 ± 0.57, *P* < 0.001), while PCB2 treatment reversed this phenomenon (Vitrified = 40.64 ± 0.57, V + PCB2 = 36.88 ± 0.59, *P* < 0.001) (Fig. 5A and C). The expression of autophagy and lysosome-related genes were further quantified. In vitrified-thawed oocytes, expression of *Beclin1* (*P* < 0.001), *Map1lc3a* (*P* < 0.01), *Ulk1* (*P* < 0.01), *Atg14* (*P* < 0.05) and *Lamp2* (*P* < 0.05) were significantly up-regulated. Notably, PCB2 treatment down-regulated the expression of *Beclin1* (*P* < 0.001), *Map1lc3a* (*P* < 0.01), *Ulk1* (*P* < 0.05) and *Lamp2* (*P* < 0.05) (Fig. 5D). DNA damage was next detected by γ-H2A.X staining (Fig. 5F). Vitrification led to a higher fluorescence intensity of γ-H2A.X (Fresh = 6.20 ± 1.51, Vitrified = 13.01 ± 1.95,* P* < 0.01), which could be rescued by PCB2 treatment (Vitrified = 13.01 ± 1.95, V + PCB2 = 6.31 ± 2.17, *P* < 0.01) (Fig. 5E).

### PCB2 has a beneficial effect on vitrified oocytes meiosis progression

**Fig. 6:**
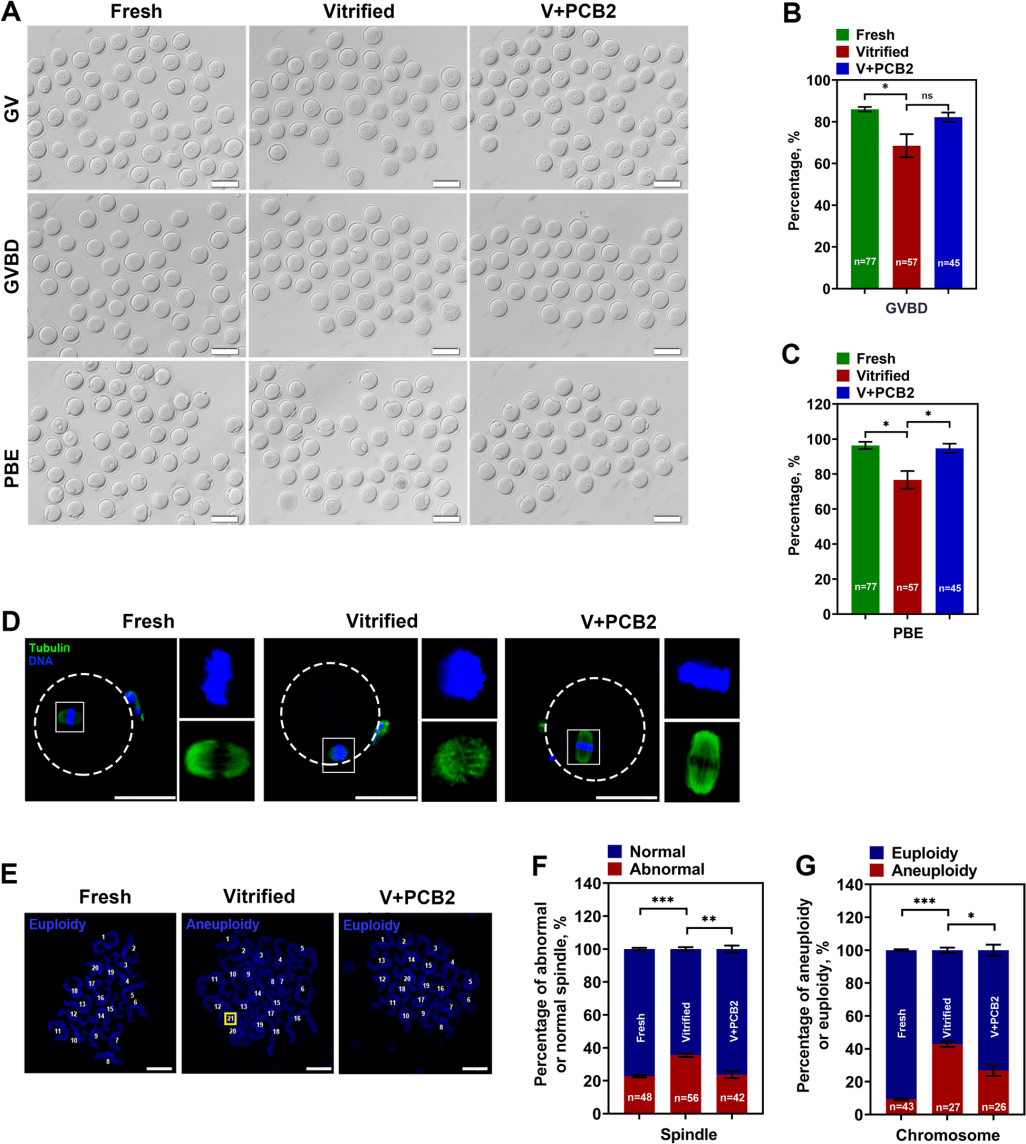
Vitrification induced meiosis progress defection was alleviated by PCB2. **A** Representative images of GV, GVBD and PBE. Scale bar = 100 μm. **B** The rate of GVBD in different groups. **C** The rate of PBE in different groups. **D** Immunofluorescent staining of matured oocytes for α-tubulin (green) and chromosome (blue). Scale bar = 50 μm. **E** Representative images of euploidy and aneuploidy chromosomes. Scale bar = 10 μm. **F** Comparison of abnormal spindle formation in different groups. **G** Rate of euploidy and aneuploidy. “n” represents the cell number used in this experiment. Data are presented as mean percentage (mean ± SEM) of at least three independent experiments. ^*^P < 0.05, ^**^P < 0.01, ^***^P < 0.001, *ns* non significance

As shown in Fig. 6A-C, GVBD (Fresh = 86.01 ± 1.10%, Vitrified = 68.54 ± 5.55%, *P* < 0.05) and PBE (Fresh = 96.34 ± 2.06%, Vitrified = 76.67 ± 5.09%, *P* < 0.05) rates were significantly decreased in vitrified oocytes compared with the fresh counterparts. To investigate whether PCB2 could alleviate meiosis damage of mouse oocytes caused by vitrification, PCB2 was added into in vitro maturation medium. PCB2 significantly increased the rate of PBE (Vitrified = 76.67 ± 5.09%, V + PCB2 = 94.66 ± 2.68%, *P* < 0.05), but had no effect on the occurrence of GVBD (Vitrified = 68.54 ± 5.55%, V + PCB2 = 82.22 ± 2.22%,* P* > 0.05). Furthermore, an increased rate of disorganized spindle apparatuses was present in vitrified oocytes, while PCB2 promoted the formation of normal spindle (Vitrified = 35.51 ± 1.10%, V + PCB2 = 23.69 ± 2.10%,* P* < 0.01) (Fig. 6D and F). The occurrence of aneuploidy in the vitrified group was rescued by PCB2 treatment (Vitrified = 42.77 ± 1.57%, V + PCB2 = 26.84 ± 3.43%,* P* < 0.05) (Fig. 6E and G).

### PCB2 affects spindle migration and F-actin density in vitrified oocytes

**Fig. 7:**
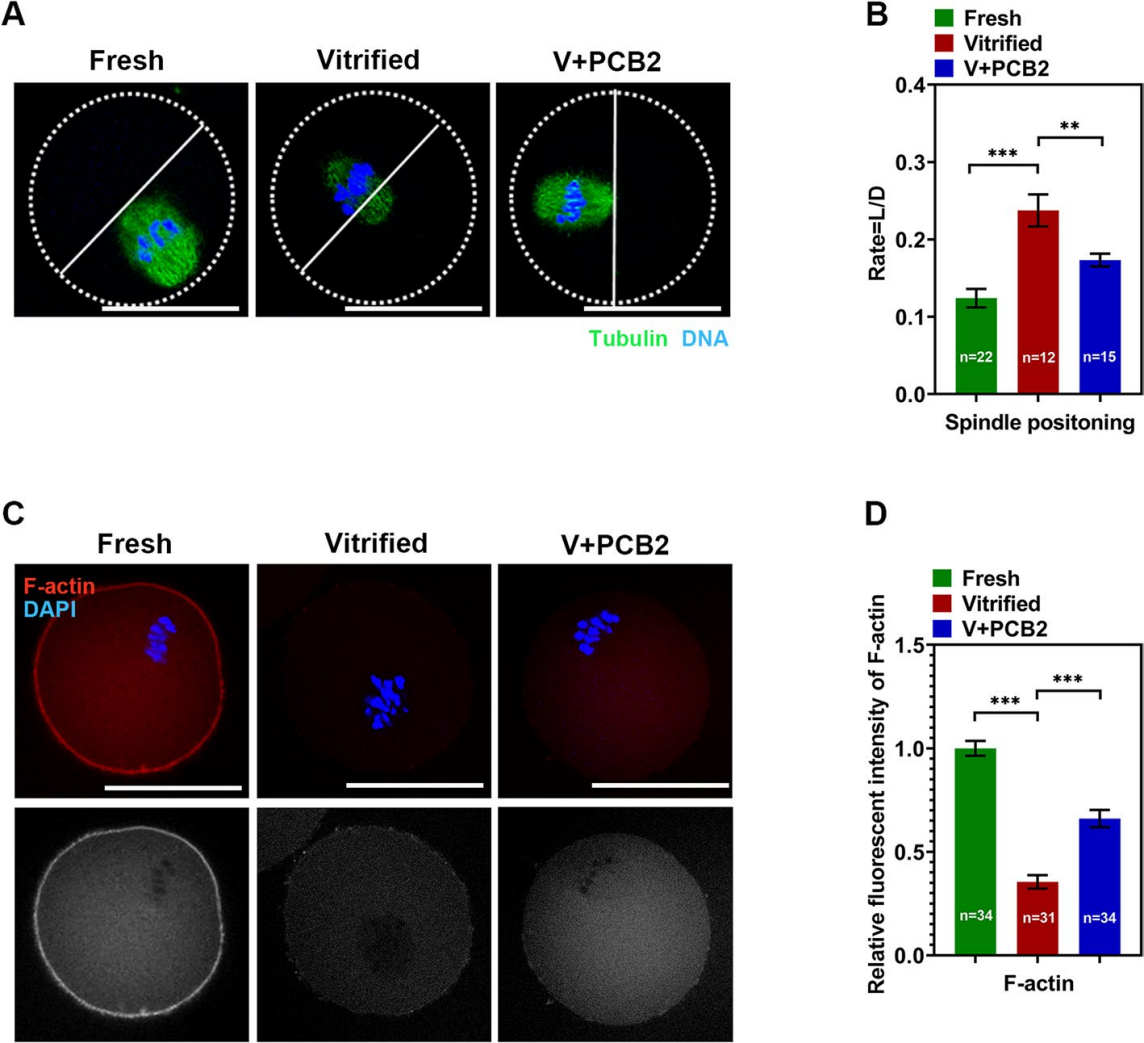
F-actin and spindle migration after vitrification. **A** The distance between the spindle pole to the cortex (Length, L) and the diameter (D) of oocyte was quantified to determine the cortically and centrally positioned spindles. Scale bar = 50 μm. **B** Rate of L/D in different groups. **C** Representative images of F-actin in different groups. Scale bar = 50 μm. **D** Quantification of F-actin fluorescence intensity. “n” represents the cell number used in this experiment. Data are presented as mean percentage (mean ± SEM) of at least three independent experiments. ^**^P < 0.01, ^***^P < 0.001

Spindle migration during oocyte maturation is critical for polar body formation. To investigate how vitrification reduces polar body extrusion, spindle position in oocytes was examined. The distance between the spindle pole to the cortex (Length, L) and the diameter (D) of the oocyte was quantified to determine the cortically and centrally positioned spindles (Fig. 7A-B). After 9 h culture of oocytes, compared to the fresh group (0.12 ± 0.01%), a large proportion of spindles in the vitrified group remained in the center of the oocytes, whereas most spindles in the fresh and PCB2 treatment group migrated to the cortex (Vitrified = 0.23 ± 0.02%, V + PCB2 = 0.17 ± 0.01%,* P* < 0.01). Since F-actin controls chromosome gathering and spindle positioning in oocytes, we next analyzed the actin filament in the MI stage to further explore the mechanism underlying spindle positioning defects. When normalized with that of the fresh group, the total F-actin fluorescent signal in the vitrified oocytes was decreased and PCB2 treatment rescued this phenomenon (Vitrified = 0.35 ± 0.03%, V + PCB2 = 0.66 ± 0.04%,* P* < 0.001) (Fig. 7C-D).

### PCB2 elevates cortical tension in vitrified oocytes

**Fig. 8:**
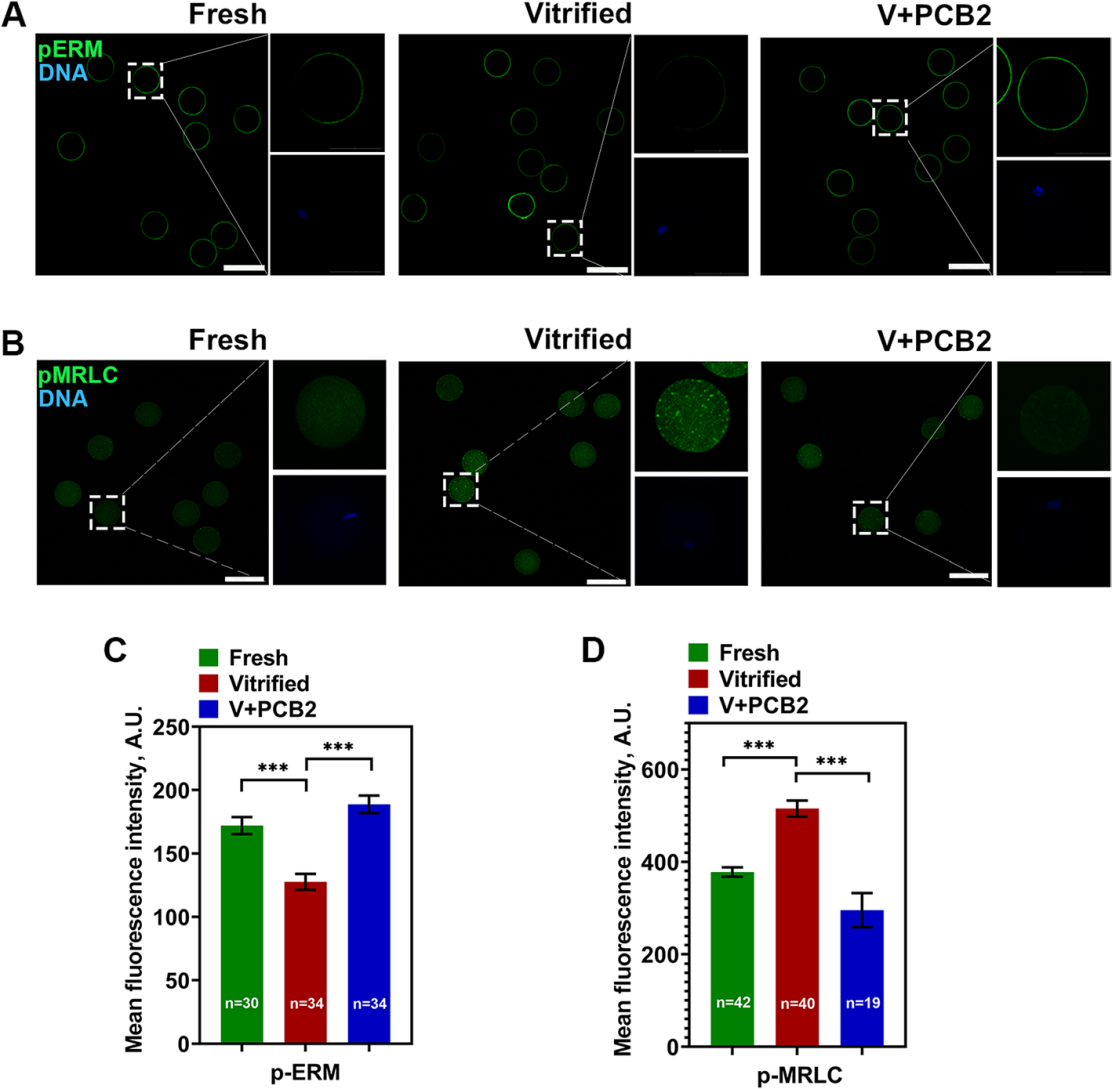
PCB2 reestablishes cortical tension in vitrified oocytes. **A** Immunofluorescent staining of matured oocytes for pERM. DNA was counterstained with DAPI (blue). Scale bar = 100 μm. **B** Immunofluorescent staining of matured oocytes for pMRLC. DNA was counterstained with DAPI (blue). Scale bar = 100 μm. **C** Mean fluorescence intensity of pERM signals. **D** Mean fluorescence intensity of pMRLC signals. “n” represents the cell number used in this experiment. Data are presented as mean percentage (mean ± SEM) of at least three independent experiments. ^***^P < 0.001

The actin cortex functions in directing spindle migration in part through membrane tension regulation. To verify whether cortical tension was altered in vitrified oocytes, pERM and pMRLC were examined. As shown in Fig. 8A-B, the fluorescent signal of pERM was significantly decreased in vitrified groups (Fresh = 172.00 ± 6.69, Vitrified = 127.70 ± 6.31, *P* < 0.001). Moreover, the fluorescent signal of pMRLC in the cytoplasm was significantly increased after vitrification (Fresh = 387.20 ± 10.05, Vitrified = 515.40 ± 17.54, *P* < 0.001). The role of PCB2 played in cortical tension regulation under vitrification stress was also explored. PCB2 treatment could restore cortical tension in vitrified oocytes as evidenced by elevated pERM (Vitrified = 127.70 ± 6.31, V + PCB2 = 188.80 ± 6.87, *P* < 0.001) and reduced pMRLC intensities (Vitrified = 515.40 ± 17.54, V + PCB2 = 295.80 ± 36.77, *P* < 0.001) (Fig. 8C-D).

### Cortical tension regulation associated with ameliorated mitochondrial function

**Fig. 9:**
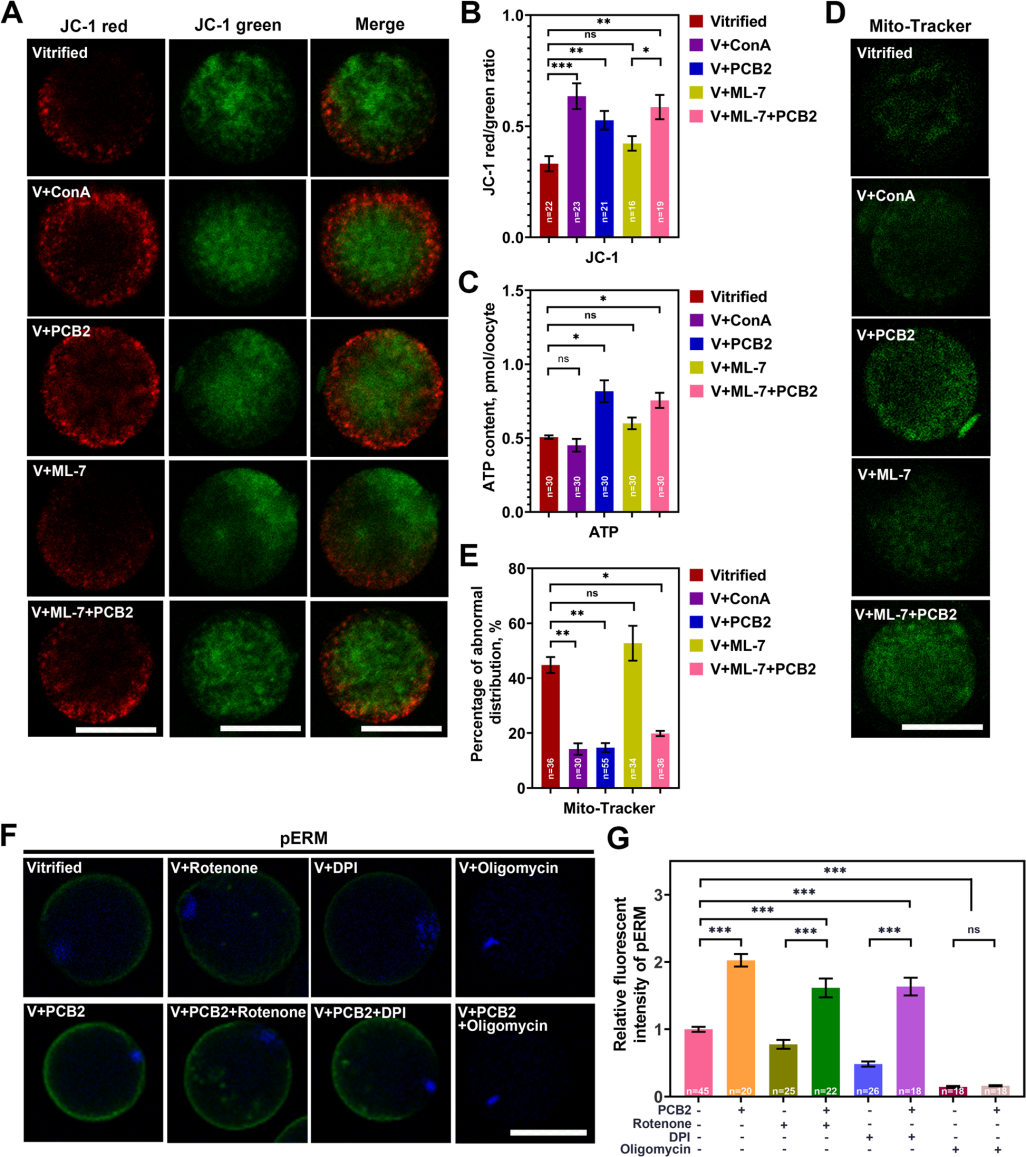
PCB2 and ConA exert different roles in mitochondrial function. **A** Mitochondrial membrane potential was detected by JC-1 staining. ConA, PCB2 and ML-7 were supplemented in recovery medium for 1 h during warming process. Scale bar = 50 μm. **B** Quantification of the MMP level in different groups. **C** ATP was measured in different groups. **D** Representative images of mitochondria distribution in different groups. Oocytes were stained with Mito-Tracker Green. Scale bar = 50 μm. **E** Rate of abnormal mitochondria distribution in different groups. **F** pERM staining of matured oocytes. DNA was counterstained with DAPI (blue). Scale bar = 50 μm. **G** Relative fluorescence intensity of pERM signals was recorded in different groups. “n” represents the cell number used in this experiment. Data are presented as mean percentage (mean ± SEM) of at least three independent experiments. ^*^P < 0.05, ^**^P < 0.01, ^***^P < 0.001, *ns* non significance

ConA is a tetravalent lectin that crosslinks the cell surface through binding to membrane glycosylated proteins [29]. It has been reported that ConA treatment can increase cortical tension in oocytes [24]. Here, we use ConA or myosin light chain kinase (MLCK) specific inhibitor ML-7 to induce increased or decreased cortical tension respectively, and further investigate their effects on MMP, mitochondrial distribution and ATP production. Compared to the vitrified control group (0.33 ± 0.03), both PCB2 (0.53 ± 0.04, *P* < 0.01) and ConA (0.64 ± 0.06,* P* < 0.001) significantly increased the MMP after vitrification (Fig. 9A-B). PCB2 also reversed MMP reduction after ML-7 treatment (*P* < 0.05). Moreover, PCB2 (14.72 ± 1.63%, *P* < 0.01), as well as ConA (14.21 ± 2.09%, *P* < 0.01), significantly alleviated the abnormal distribution (Fig. 9D-E). Interestingly, unlike ConA (0.45 ± 0.04 pmol/oocyte,* P* > 0.05), PCB2 (0.82 ± 0.07 pmol/oocyte, *P* < 0.05) also exhibited an additional role in promoting ATP production compared with vitrified group (0.51 ± 0.01 pmol/oocyte) (Fig. 9C). The results showed that PCB2 treatment not only alleviated mitochondrial defects but also promoted ATP production. This drove us to further investigate the interplay between cortical tension and ATP. Thus, vitrified oocytes were treated with rotenone (mitochondrial respiratory chain complex I inhibitor), diphenyleneiodonium (DPI, pentose phosphate pathway inhibitor), and oligomycin (ATP synthase inhibitor). The expression of pERM was significantly decreased after the treatment of the three inhibitors (*P* < 0.001) (Fig. 9F). Among them, oligomycin almost eliminated pERM expression (Fig. 9G). Vitrified oocytes were then treated with PCB2 in combination with the individual inhibitor mentioned above. Results showed that PCB2 could rescue the cortical tension reduction induced by rotenone or DPI (*P* < 0.001), but not oligomycin (*P* > 0.05) (Fig. 9G). This indicated that PCB2 mediated cortical tension through the electron transport chain and pentose phosphate pathway.

### PCB2 was mainly involved in the regulation of glycometabolism during embryo development

**Fig. 10:**
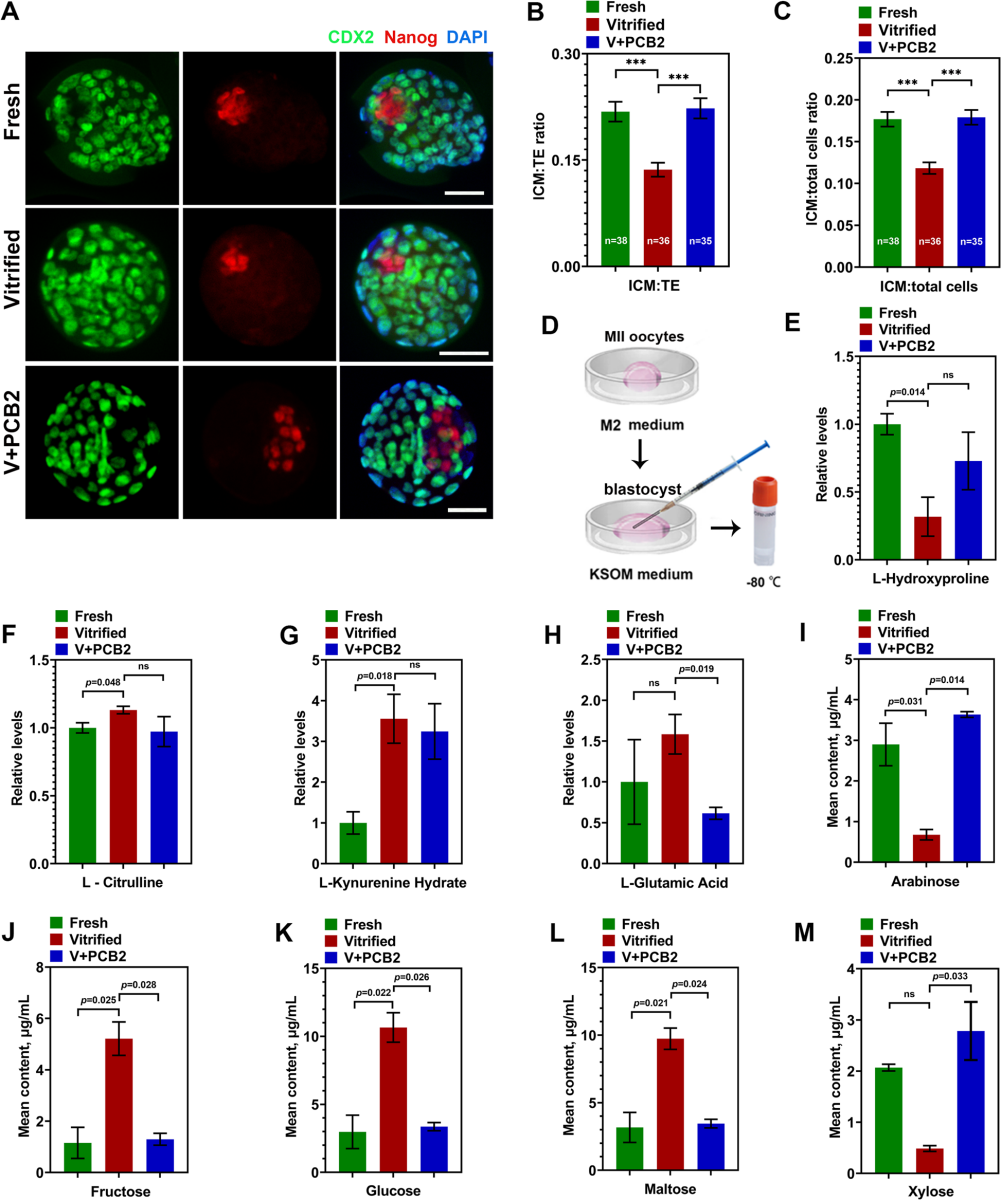
PCB2 was mainly involved in the regulation of glycometabolism during embryo development. **A** Immunofluorescent staining of CDX2 and Nanog in blastocysts from different groups. DNA was counterstained with DAPI (blue). Scale bar = 50 μm. **B**-**C** The ICM: TE rate and ICM: total cells rate were compared in different groups. **D** Schematic diagram of metabolomic sample collection. **E**–**H** The relative contents of four amino acids (L-hydroxyproline, L-citrulline, L-kynurenine hydrate, and L-glutamic acid) in different groups. **I**-**M** The mean contents of five carbohydrates (arabinose, fructose, glucose, maltose, and xylose) in the medium of different groups. “n” represents the cell number used in this experiment. Data are presented as mean percentage (mean ± SEM) of at least three independent experiments. ^***^P < 0.001, *ns* non significance

As shown in Fig. 10A-C, the blastocyst quality was greatly improved after PCB2 treatment indicated by CDX2 (a cell lineage-specific marker for trophectoderm (TE)) and Nanog (a cell lineage-specific marker for inner cell mass (ICM)) staining. Both the ratio of ICM to TE (*P* < 0.001) and ICM to total cell number (*P* < 0.001) were significantly increased in the PCB2 group. To further dissect the underlying mechanisms of the effects of PCB2 on the subsequent embryo development of vitrified oocytes, we performed targeted metabolomics analysis of day4 culture media from fresh, vitrified and PCB2 treatment groups. PCB2 treatment significantly affected the metabolism of five saccharides, while only one amino acid was affected (Fig. 10D-M). This implied that PCB2 was mainly involved in glycometabolism to regulate embryonic development.

### PBC2 regulates cortical tension in morula and blastocyst cells after vitrification

**Fig. 11:**
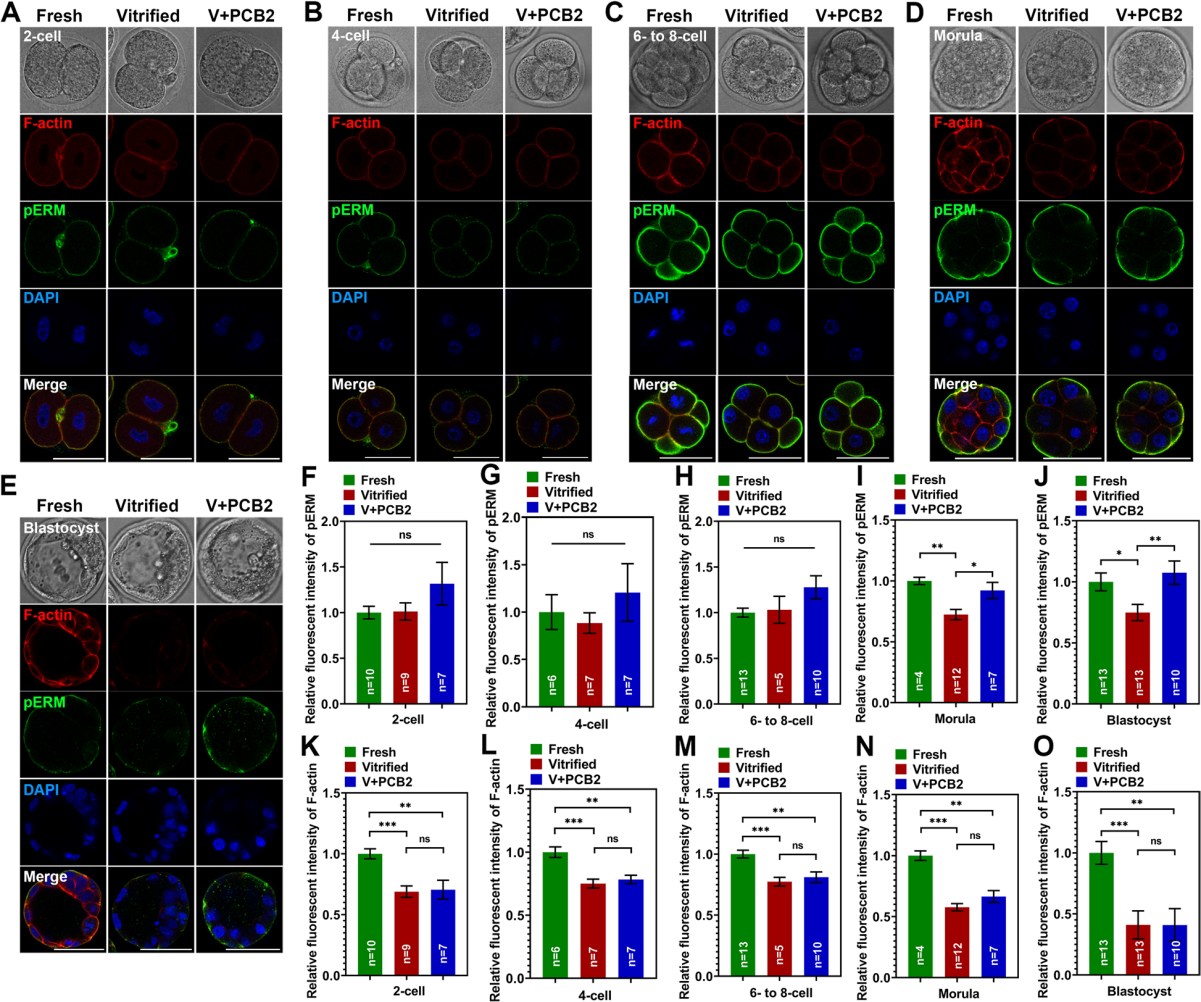
PCB2 has a long-lasting effect on the cortical tension of embryos. **A**-**E** Immunofluorescent staining of pERM in different stages of embryo. Scale bar = 50 μm. **F**-**J** Relative fluorescence intensity of pERM signals was recorded in different groups. **K**–**O** Quantification of F-actin fluorescence intensity.“n” represents the cell number used in this experiment. Data are presented as mean percentage (mean ± SEM) of at least three independent experiments. ^*^P < 0.05, ^**^P < 0.01, ^***^P < 0.001, *ns* non significance

pERM was used as an indicator to investigate the change of cortical tension in embryo cells from different stages after vitrification. As shown in Fig. 11, cell cortical tension in 2-cell, 4-cell and 6- to 8-cell embryos was not affected after vitrification, but cell cortical tension significantly decreased at morula and blastocyst stages. PCB2 can improve the cell cortical tension abnormalities caused by vitrification. Interestingly, the fluorescent signal of F-actin in embryo cells was significantly reduced in the vitrified group (*P* < 0.001), and PCB2 did not play a positive role (*P* > 0.05).

## Discussion

In the present study, the mechanism by which the natural antioxidant PCB2 improved the viability of vitrified oocytes was revealed, and the interaction between cortical tension and mitochondrial function during vitrification was investigated. To our knowledge, this is the first report to unravel increased cortical tension as another contributor for improved viability in vitrified oocytes and clarify the muti-protective roles of PCB2 in response to vitrification stimuli.

Cryopreservation is a method of choice for establishing animal gene banks and preserving female fertility. However, MII oocyte cryopreservation is much more challenging among reproductive cells and tissues, mainly because of the large size, low surface area to volume ratio, relatively high water content, and presence of the meiotic spindle in MII oocytes [58]. It is reported that vitrification can induce the generation of excessive ROS, which severely impairs endogenous antioxidant systems in oocytes [55]. As a powerful antioxidant, procyanidin extract provides significantly greater protection against free radicals and free radical-induced lipid peroxidation and DNA damage than vitamins C, E [59]. PCB2, a member of oligomeric anthocyanins precursors, balanced the redox status of vitrified oocytes and significantly improved mitochondrial function (Figs. 2 and 3). Mitochondria also have multiple functions, including the regulation of calcium and actively participating in the regulation of signal transduction pathways [60]. Vitrification triggered [Ca^2+^]_ER_ release leading to abnormally increased [Ca^2+^]_m_ levels in bovine [61], which was also confirmed in our results (Fig. 4). The accumulation of ROS can further cause double-strand breaks (DSB) [62]. Early apoptosis and autophagy were also alleviated through PCB2 antioxidant property (Fig. 5). These results confirmed that PCB2 can restore intracellular calcium homeostasis and redox levels through regulating mitochondrial function.

The damage caused by vitrification to the meiotic process is directly reflected in the two processes of germinal vesicle breakdown and pole body extrusion [63]. PCB2 can rescue the decreased PBE rate in vitrified oocytes (Fig. 6). Although vitrified oocytes can extrude the first polar body after meiosis resumption, the chromosome alignment and spindle formation are more error-prone [64]. PCB2 not only played a positive role in spindle formation, but also significantly reduced the aneuploidy rate of vitrified oocytes. The functional integrity of the spindle is also demonstrated by the migration from the oocyte center to the cortex during the MI phase, which is mediated by F-actin [65]. Disruption of F-actin also contributes to the generation of aneuploid oocytes [66]. As expected, PCB2 ameliorated the MI-phase spindle migration damage caused by vitrification and increased the F-actin signal intensity (Fig. 7). These results indicated that PCB2 can upregulate vitrified oocyte quality by restoring the meiotic process.

Changes in cell fate are often accompanied by changes in cell shape and mechanics. Oocyte underwent a dramatic osmotic pressure change during the vitrification/thawing process, which resulted in a drastic change in the morphology of the oocyte [67, 68]. Cortical tension is mediated by ERM family, myosin-II and actin [19]. In the present study, the level of pERM, which was positively correlated with cortical tension [24], significantly decreased in the vitrified oocyte (Fig. 8). The change of pMRLC after vitrification was also consistent with the cortical tension reduced model [28, 29]. However, PCB2 showed the same effect as ConA, which helps to re-establish cortical tension (pERM and pMRLC) (Fig. 8).

Cytoskeletal dynamics induced by mechanical signals can induce cytoplasmic enzyme response and/or activity to influence cell metabolism [69, 70], and also play a critical role in the regulation of mitochondrial structure [71, 72]. Therefore, changes in cortical tension are likely to be related to mitochondrial function. PCB2 not only rescued the decline of MMP like ConA, but also promoted ATP production, which implied that PCB2 had an extra role. However, there was a discrepancy between MMP and ATP content, previous finding also discovered that native mitochondrial ATP (ATPmito) and mitochondrial inner membrane potential (IMPmito) were not necessarily correlated under physiological conditions [73]. This can be interpreted as IMPmito at any given time is simply the difference in voltage in the mitochondrial inner membrane, but ATPmito is a result of not only production but also consumption and flux from the mitochondrial matrix [73]. Furthermore, PCB2 corrected the abnormal mitochondrial distribution (Fig. 9). The results indicate that the mechanism underlying PCB2 modulated cortical tension elevation differs from that of ConA. The cortical tension increased by ConA can only affect mitochondrial localization and inner membrane voltage from the cytoskeleton dynamics, while the antioxidant PCB2 probably participates in the consumption and flux of ATP in the mitochondrial matrix in addition to the above functions.

Mitochondria provide ATP through electron transport chain coupling OXPHOS, which has been proved necessary for cytoskeletal migration [74, 75]. For example, myosin is an ATP-dependent actin-based molecular motor that performs a variety of functions such as spindle assembly, spindle orientation, chromosome segregation, and cytokinesis [76]. Rotenone induces free radical formation, ATP production deficiency and impairs oocyte maturation by inhibiting mitochondrial electron transport chain complex I [77, 78]. Oligomycin binds to the mitochondrial membrane embedding area F0 and blocks proton conductance through the inner membrane, thereby inhibiting the synthesis and hydrolysis of ATP [79]. Rotenone and oligomycin both severely induce decreased cortical tension in vitrified oocytes. However, PCB2 could rescue the inhibition of rotenone, but not oligomycin (Fig. 9). Pentose phosphate pathway (PPP) inhibitor DPI was also used to determine the effect of metabolism on cortical tension in vitrified oocytes. DPI inhibits NADPH oxidase, which is an enzyme that produces NADP required for PPP activity [80]. DPI treatment of porcine oocytes can inhibit glycolysis and PPP, resulting in decreased intracellular glutathione concentration and maturation rate [81]. PCB2 also salvaged the decline in cortical tension caused by DPI (Fig. 9). The above results indicate that PCB2 could influence ATP flux through the electron transport chain, but PCB2 loses function to cortical tension when ATP synthesis is blocked. Further proved that ATP production directly affects cortical tension.

Since PCB2 has a positive effect on vitrified oocyte viability, we then explored the role in embryo development. The increased ICM ratio was always correlated with high developmental potential [82]. As expected, PCB2 enhanced the quality of subsequent blastocyst, indicated by increased ICM cell number (Fig. 10). Before implantation, glucose uptake and utilization were prerequisites for embryo survival and normal development, abnormal glucose transport resulted in programmed embryonic death [83]. Hence, sufficient and timely glucose transport was important in maintaining the dynamic balance of glucose metabolism in oocytes and embryos [84, 85]. Vitrification decreased glucose transporter 1 (GLUT1) expression in mouse MII oocytes, leading to abnormal glucose transport and metabolism [57], which was further verified by our targeted metabolomics analysis of day 4 culture medium (Fig. 10D). PCB2 treatment rescued metabolism deficiency by restoring glycometabolism activity in embryos. PPP is important in oocytes glycometabolism [86] and controls TE cell fate [87]. These results further demonstrate that PCB2 could relieve the effect of PPP inhibitor DPI on oocytes, and promote embryo development [88]. Thus, our data imply that PCB2 has a significant protective effect on vitrified oocytes and subsequent embryo development through glycometabolism regulation.

Embryo mechanical property is also an important indicator to evaluate it’s quality [89]. The superior effects of PCB2 on oocytes can extend into embryonic development, which prompted us to further explore whether PCB2 is involved in the mediation of cortical tension in the embryo cells. During blastocyst development, the lumenal pressure increases about twofold, which translates into a concomitant increase in cell cortical tension and tissue stiffness of the trophectoderm [90]. In the present study, the cortical tension of embryo cells in different stages was detected by pERM immunofluorescence staining. Results showed that vitrification did not alter cortical tension in embryo cells at the 2-cell to 6–8-cell stages, but significantly reduced cortical tension in morula and blastocyst cells. Our results implied that vitrification-induced decreased cortical tension would impact further embryo development, which was consistent with the previous finding that the reduced pressure would lead to the decreased developmental potential of thawing embryos [91]. It was reported that during embryo development, cortical F-actin ring assembles at the apical pole of the embryo’s outer cells, subsequently forming a ring structure and extending to the cell–cell junction and initiating a tension-dependent zipper mechanism along the junction, which is required to seal the embryo for blastocyst formation [92]. In our study, the decreased cortical tension of embryo cells was observed at the morula stage, which indicated that the development capacity for blastocyst formation was compromised. As expected, PCB2 contributes to the re-establishment of cortical tension in morula and blastocyst embryo cells, suggesting that PCB2 also plays an active role in embryonic development in addition to regulating metabolism.

## Conclusion

The present study unravels the additional role of antioxidant PCB2 in promoting oocyte quality through mitochondrial ATP production regulated cortical tension. Our results will provide an integrative perspective into understanding the cryoinjuries in oocytes, and contribute to clarifying the protective roles of polyphenolic compounds in cortical tension during the vitrification/thawing process.

## Supplementary Information

**Additional file 1: Table S1.** Mass spectrometry multi reaction monitoring (MRM) collection parameters.

## Data Availability

All data generated or analyzed during this study are included in this published article.

## Abbreviations

PCB2: Procyanidin B2
MII: Metaphase II
ERM: Ezrin/Radixin/Moesin family
pERM: Phosphorylated-ERMs
pMRLC: Phosphorylated-myosin-II light chain
OXPHOS: Oxidative phosphorylation
ROS: Reactive oxygen species
GV: Germinal vesicle
GVBD: Germinal vesicle breakdown
PBE: Polar body extrusion
ConA: Concanavalin A
HTF: Human tubal fluid
DMSO: Dimethylsulfoxide
EG: Ethylene glycol
IF: Immunofluorescence
PFA: Paraformaldehyde
DAPI: 4′,6-Diamidino-2-phenylindole
MMP: Mitochondrial membrane potential
[Ca^2+^]_m_: Mitochondrial calcium
[Ca^2+^]_ER_: Endoplasmic Reticulum calcium
qPCR: Quantitative polymerase chain reaction
UPLC: Ultra-performance liquid chromatography
MTY: Mito Thermo Yellow
MI: Metaphse I
MLCK: Myosin light chain kinase
DPI: Diphenyleneiodonium
TE: Trophectoderm
ICM: Inner cell mass
DSB: Double-strand breaks
ATPmito: Mitochondrial ATP
IMPmito: Mitochondrial inner membrane potential
PPP: Pentose phosphate pathway
