# Supplementary material for: Antioxidant procyanidin B2 protects oocytes against cryoinjuries via mitochondria regulated cortical tension
Source: J Anim Sci Biotechnol. 2022 Aug 16;13:95. doi: 10.1186/s40104-022-00742-y (PMC9380387; doi:10.1186/s40104-022-00742-y)
Supplement: Supplementary file 1 — Additional file 1: Table S1. Mass spectrometry multi reaction monitoring (MRM) collection parameters. [file 40104_2022_742_MOESM1_ESM.docx]

**Supplemental Information: Additional file 1**

| **Table S1.** Mass spectrometry multi reaction monitoring (MRM) collection parameters | | | |
| --- | --- | --- | --- |
| Metabolites | DP, V | CE, eV | RT, min |
| Serine | 90 | 15 | 8.11 |
| Alanine | 90 | 13 | 7.83 |
| Dimethylglycine | 90 | 21 | 8.06 |
| Proline | 90 | 13 | 7.52 |
| Valine | 90 | 13 | 7.5 |
| Threonine | 90 | 13 | 7.95 |
| DL-Pipecolic acid | 90 | 18 | 7.75 |
| Hydroxyproline | 90 | 19 | 7.51 |
| Leucine-isoleucine | 90 | 13 | 7.12 |
| Ornithine | 90 | 14 | 10 |
| Aspartate | 90 | 17 | 7.83 |
| Lysine | 90 | 32 | 10.1 |
| Methionine | 90 | 12 | 7.25 |
| Histidine | 90 | 14 | 9.94 |
| Phenylalanine | 90 | 30 | 6.95 |
| 1-Methyl-Histidine | 90 | 20 | 9.73 |
| Arginine | 90 | 16 | 10.2 |
| Citrulline | 90 | 14 | 8.17 |
| Tyrosine | 90 | 39 | 7.41 |
| Acetyllysine | 90 | 23 | 7.69 |
| N-acetyl-glutamate | 90 | 24 | 5.03 |
| Tryptophan | 90 | 18 | 7.05 |
| Kynurenine | 90 | 25 | 7.13 |
| Cystine | 90 | 32 | 9.04 |
| Sarcosine | 90 | 20 | 7.79 |
| Glycine | 90 | 18 | 4.83 |
| Homoserine | 90 | 32 | 7.95 |
| Glutamate | 90 | 17 | 5.02 |
| 3-Phospho-serine | 90 | 12 | 7.38 |
| S-adenosyl-L-methionine | 90 | 15 | 10.9 |
| Isoleucine | 90 | 13 | 7.1 |
| Homocysteine | 90 | 17 | 9.17 |
| Pyroglutamic acid | -90 | -19 | 5.11 |
| N-Acetyl-L-alanine | -90 | -14 | 4.75 |
| Hydroxyisocaproic acid | -90 | -16 | 3.72 |
| S-adenosyl-L-homocysteine | -90 | -31 | 9.5 |
| Pyruvate | -44 | -11 | 3.48 |

Abbreviation: *DP*, declustering potential; *CE*, collision energy; *RT*, retention time
